# Postvaccination Influenza 2009 H1N1 Respiratory Failure Requiring Extracorporeal Membrane Oxygenation

**DOI:** 10.1155/2011/167963

**Published:** 2011-10-29

**Authors:** Michael S. Firstenberg, Erik Abel, Danielle Blais, Juan Crestanello, Julie E. Mangino

**Affiliations:** ^1^Division of Cardiac Surgery, The Ohio State University Medical Center, Columbus, OH 43210, USA; ^2^Division of Infectious Diseases, The Ohio State University Medical Center, Columbus, OH 43210, USA

## Abstract

The spread of pandemic Influenza A (H1N1-2009) was believed to have been attenuated by the effectiveness of worldwide vaccination initiatives. Despite the immunogenicity of a safe vaccine, we report a case of vaccine failure resulting in catastrophic influenza-associated respiratory failure.

 Pandemic Influenza A (H1N1-2009) was not only distinguished by a predilection towards relatively young and otherwise healthy patients, but was also associated with a high incidence of catastrophic respiratory failure requiring extracorporeal membrane oxygenation (ECMO) [1, 2]. The rapid development and distribution of an effective vaccine was attributed to attenuating what was already a pandemic outbreak. Nevertheless, even in 2011, H1N1-2009 continues to be a challenging clinical and epidemiologic problem. 

H1N1-2009 vaccination was determined to be >95% effective within 3 weeks in otherwise healthy adults [3, 4]. While *serious* infections in vaccinated patients have been reported [5], many of these patients have risk factors for vaccination failure—such as chronic institutionalization or disabled and debilitated patients who may lack a functional immune system—and the “severity” of illness is debatable. We present a case of a previously healthy male, with documented vaccination and a sufficient lag time to develop an appropriate antibody response, in which severe H1N1-2009-associated respiratory failure developed requiring ECMO.

Our patient was a 53-year old previously healthy, nonsmoker with no reported unusual recent exposures, male who presented to an outside hospital with severe respiratory distress. He was started on empiric broad-spectrum antibiotics and oseltamivir phosphate (150 mg PO BID, Tamiflu, Genentech, Inc., San Francisco, Calif, USA) and Methylprednisolone (60 mg intravenous, Q6H for 3 days). Initial bacterial blood and respiratory cultures were negative, but had a positive nasal swab for Influenza H1N1. Initial chest X-ray showed severe diffuse pulmonary infiltrates and edema consistent with adult respiratory distress syndrome (ARDS, Figure 1). Five days later, due to failure of maximal mechanical ventilation and worsening hypoxemia with hypercarbia he was placed on percutaneous veno-veno ECMO and transferred to our Institution. Upon arrival, bacterial and fungal blood, urine, and respiratory cultures from a bronchial alveolar lavage were obtained and were negative. Real-time polymerase chain reaction assay was positive for H1N1-2009. Other viral cultures, including HIV-1/-2 and a hepatitis panel, were negative. A transthoracic echocardiogram was unremarkable. Over several days, antibiotic therapy was deescalated, and he completed a 10-day course of oseltamivir.

After 7 days of ECMO support, he was successfully weaned and decannulated. His ventilator requirements slowly improved, and despite an *Enterobacter aerogenes* ventilator-associated pneumonia that developed post-ECMO decannulation, the remainder of his hospital course was unremarkable. He was discharged for rehabilitation 22 days after ECMO decannulation (hospital day 31).

At followup, 4 months following-discharge, he was doing well, repeat chest X-ray showed no evidence of acute or chronic disease, and pulmonary function testing showed normal spirometry, a mild restrictive ventilatory defect, a mildly impaired diffusing capacity (23.6 ml/min/mmHg, 74% predicted), and a normal 6-minute walk (515 meters, normal: 365 meters). In discussion with the patient, he recalled having been vaccinated against “the flu”. Following appropriate release of medical records, it was confirmed that 2 months prior to the onset of symptoms he received 0.5 ml of inactivated trivalent influenza vaccine in the left deltoid (Fluzone, Sanofi-Pasteur Inc., Swiftwater, Pa, USA) [6]. Vaccination was provided by a local Pharmacy run vaccination clinic with established policies and procedures conforming to State and Federal regulations regarding the storage and administration of vaccines. No postvaccination problems were reported. 

Influenza H1N1-2009 was a formidable challenge worldwide during 2009 in terms of severity of illness and ease of transmission, particularly in otherwise young and healthy patients [7]. The Australian experience estimated 1.6–2.6 cases per million required ECMO following infection with H1N1. In the 68 patients who required ECMO the mortality was 21%—a rate much greater than expected with recent seasonal Influenza outbreaks [2]. 

Large-scale vaccination efforts were undertaken to minimize the spread and impact of the evolving pandemic. While the immunogenicity of the vaccine has been documented, the incidence of seroconversion is variable. In one study, only 75% of adults seroconverted [8] while another study demonstrated a 95% seroconversation rate within 21 days in those adults who received a single 15 ug dose of a trivalent, unadjuvanted, inactivated, split-virus vaccine [4]. Regardless, given inherent variability in antigenicities and immune responses, with such a large population at risk, even a small percentage of vaccine failure can have a significant epidemiologic impact. Furthermore, considering the virulence of H1N1-2009 the risk of developing catastrophic respiratory failure requiring high-intensity ventilator or even ECMO support is not negligible.

**Conclusions**

Our recent case, in a patient who presented in early 2011, illustrates several concerns. First, despite less worldwide emphasis, seasonal flu, and in particular H1N1, remains a significant and potentially deadly problem in relatively young and healthy patients. More importantly, clinicians who encounter patients with severe “influenza like illnesses” even with documented vaccine histories should strongly be considered for appropriate antiviral therapies. In severe cases, ECMO should be considered and can be life saving in those patient failing conventional ventilator therapies [9].

##  Conflict of Interests

Dr Michael Firstenberg (First Author) serves as a scientific advisor to Maquet Cardiovascular LLC, the manufacturer of the oxygenator component of the ECMO circuit, and has received modest financial support (<$10,000) for these activities. None of the other authors have any conflicts of interests to report in the context of this paper.

## Figures and Tables

**Figure 1:**
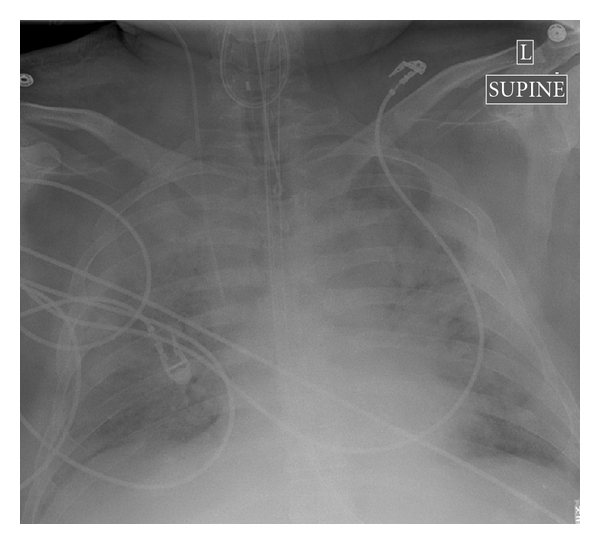
Admission chest X-ray showing severe bilateral diffuse pulmonary infiltrates.
